# OP12 Identification Of Patient And Societal Needs In Melanoma: Application Of The Needs Examination, Evaluation And Dissemination (NEED) Assessment Framework

**DOI:** 10.1017/S026646232400076X

**Published:** 2025-01-07

**Authors:** Claudia Schönborn, Mats De Jaeger, Muriel Levy, Laurence Kohn, Rani Claerman, Robby De Pauw, Irina Cleemput, Charline Maertens de Noordhout

## Abstract

**Introduction:**

The NEED (Needs Examination, Evaluation and Dissemination) assessment framework aims to identify patient and societal unmet needs in various diseases, highlighting gaps for intervention. This approach is exemplified through a malignant melanoma case study, ranking sixth in European cancers. Therapeutic progress has been made over the last decade, prompting an assessment of remaining unmet needs.

**Methods:**

The applicability of the NEED framework has been evaluated through a case study of melanoma in Belgium. The methodology had four components: (i) an overall description of the disease based on the scientific (published and grey) literature and clinical expert input; (ii) an online survey on the unmet needs of patients (n=37); (iii) semistructured interviews with individuals affected by melanoma (n=9); and (iv) secondary data collection for each NEED framework criterion, including scientific literature and public databases. The survey results were analyzed with descriptive statistics, and the interview transcripts were analyzed using a thematic analysis.

**Results:**

Unmet needs in melanoma were categorized as patient-level health, healthcare, and social needs, alongside wider societal needs. Patients experienced a substantial burden from mortality in advanced stages, burdensome symptoms such as fatigue, and notable side effects of systemic treatment. Inadequate psychological support contributed to high levels of fear and anxiety. A scarcity of dermatologists posed a barrier to timely care access, and low awareness of melanoma risk led many patients to delay seeking medical attention, with significant societal implications for melanoma prevention efforts. The case study also identified research gaps concerning social and ethnic inequalities in melanoma care in Europe.

**Conclusions:**

Using the NEED framework highlighted melanoma patients’ most crucial unmet needs. This evidence can be used to assess whether new interventions address the highest unmet patient or societal needs. Challenges in ensuring adequate sample size limit the generalizability of results. Capturing melanoma’s complexity in a snapshot masks some needs heterogeneity.

